# Sleep and Cancer

**DOI:** 10.3390/cancers17060911

**Published:** 2025-03-07

**Authors:** Courtney M. Vaughn, Bradley V. Vaughn

**Affiliations:** 1 Department of Pediatrics, Division of Pediatric Hematology and Oncology, Washington University School of Medicine, St. Louis, MO 63110, USA; cmvaughn@wustl.edu; 2 Department of Neurology, Division of Sleep Medicine, University of North Carolina, Chapel Hill, NC 27599, USA

**Keywords:** sleep, sleep disorders, sleep disruption, cancer, insomnia, excessive sleepiness

## Abstract

**Background/Objectives**: Sleep issues are common in the general population, and these problems occur even more frequently for people with cancer. Sleep problems may pre-exist a patient’s cancer diagnosis, and there is a growing interest in understanding the impact of sleep on cancer development and progression. Sleep disorders may impact cancer through altered metabolism, impacts on immune response, and alterations in hormones and gene expression. Sleep disorders may also arise after, or be aggravated by, an individual’s cancer and cancer treatment. Treating a person with cancer’s sleep disorder may help improve their healing, mental health, cognition, and overall resilience. **Methods:** Studies examining a variety of aspects of the relationship between sleep and cancer were found by searching the National Library of Medicine and characterized by their specific information provided on the relationship between sleep and cancer. **Results:** This review article summarizes our current understanding of the complex inter-relationship between sleep and cancer, the underlying mechanisms that create these connections, and the methods and impact of treating sleep issues in cancer patients. The article also outlines an approach to sleep complaints for clinicians caring for patients with cancer. **Conclusions:** Significant research is still needed to understand the full relationship between sleep disorders and cancer. The impact of sleep issues on cancer and of cancer on sleep appears to be specific to the tissue and the molecular type of cancer. The treatment of sleep disorders is multimodal, and offers a promising avenue to improve the health and quality of life of cancer patients.

## Simple Summary

Over two-thirds of patients with cancer have an issue with their sleep, and some research indicates that sleep may impact the development and progression of cancer. The aim of this review is to highlight the current information on sleep issues, such as sleep duration, sleep disruption, and sleep disorders, and the development and progression of cancer, as well as the impact of cancer on sleep. This article outlines the common sleep complaints in patients with cancer and discusses a logical approach and treatment options for sleep issues in patients with cancer.

## 1. Introduction

### 1.1. What Is Sleep?

**Figure 1:**
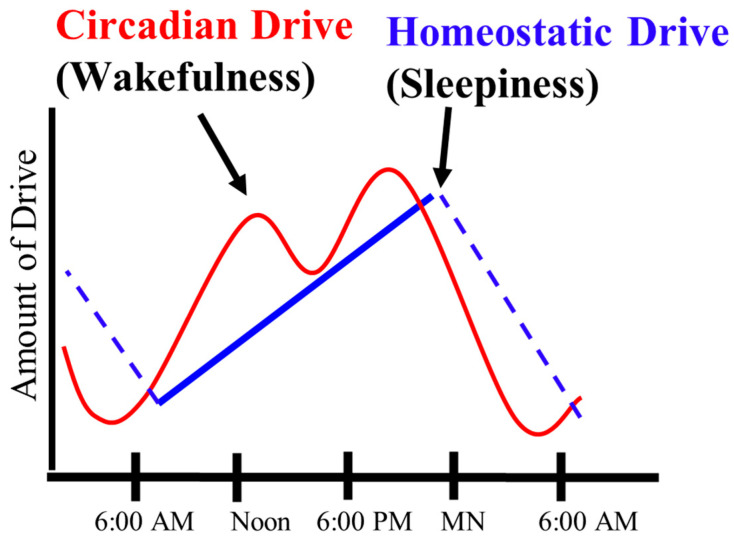
This figure depicts the two process model. The homeostatic drive increases as a result of activity while awake and then is metabolized during sleep. This drive increases the drive for sleep. The circadian drive also increases through the day and opposes the homeostatic drive by promoting wakefulness. The circadian rhythm is accentuated by light, activity, food, and social interaction during the waking period.

Sleep is a normal dynamic physiological state, which is characterized by the decreased responsiveness of the central nervous system to the environment. During this state, several brain and body functions occur that are important in the maintenance of organs and maximizing performance. Sleep is regulated, in part, by the accumulation of byproducts from neuronal and somatic activity, the homeostatic drive, and by an individual’s circadian rhythm that helps with timing of sleep periods and coordinating other cycles [1]. A variety of substances constitute the homeostatic drive; these substances increase during the wake period. These substances include, but are not limited to, inflammatory-related molecules such as adenosine, galanin, oleamide, cytokines, and tumor necrosis factor, as well as other byproducts of cellular activity. The circadian rhythm is a recurrent transcription-translation loop that creates a clock of approximately 24 h that regulates multiple physiological cycles. The control of these multiple cycles is in an attempt to optimize our preparedness for recurring physiological events and save energy when these processes are not needed. Although we think of sleep as one of these events, many processes are governed by a circadian rhythm, and over 20% of our genes are transcribed in a circadian fashion. From a sleep–wake viewpoint, the main function of the circadian rhythm is to promote our ability to be awake. The circadian drive typically builds through the day to remain awake and offset the promotion of sleep by the homeostatic drive. These two major drivers help explain the timing of sleep and the length of sleep through what is known as the two process model [2]. In this model, the circadian drive promotes wakefulness through the day, while at the same time, the homeostatic drive builds up as a chemical capacitor to promote sleep (Figure 1). Once the homeostatic drive is above the circadian drive to be awake, the person feels sleepy. Through the night, the substances comprising the homeostatic drive are metabolized and reprocessed via the mechanisms in sleep. By morning, the homeostatic drive to sleep dips below the circadian drive to be awake, and the person awakens [2].

Sleep may be contextually thought of as one state, but comprises multiple different states. Sleep is typically subdivided into non-rapid eye movement (NREM) sleep and rapid eye movement (REM) sleep [3]. NREM sleep is also subdivided into Stages N1, N2, and N3, which are associated with a gradual decrease in responsiveness to the environment and isolate the cerebral cortex from sensory inputs for processes such as replenishing neurotransmitters and energy stores, synaptic pruning, and processing flushing of waste products. Some of these functions also occur in REM sleep. We associate this state with dreaming and emotional processing. REM sleep is also thought to have important secondary effects on the immune system, autonomic system regulation, and the processing of emotions [4].

### 1.2. What Is Cancer?

Cancer is a collection of diseases linked by the characteristics of the uncontrolled growth of abnormal cells. According to the World Health Organization, approximately one in five individuals will develop cancer in their lifetime, and there are an estimated 20 million new cancer cases diagnosed and 9.7 million deaths related to cancer each year [5]. According to the American Cancer Society, more than 2 million people are diagnosed with cancer in the United States annually. As therapies have improved, the growing population of cancer survivors is estimated to be over 18 million people. These patients will require evaluation for and management of the sequelae of their disease and treatment [6]. While the different kinds of cancer share the commonality of uncontrolled cellular growth, the underlying triggers and downstream effects are unique. Any organ system can develop cancer. Additionally, cancer cells can spread to other organs, either through local invasion or by metastasis. Some of the cellular processes that allow for the spread of the cancer appear to be linked to sleep and the circadian rhythm. Significant debate exists as to the impact of sleep on cancer risk and progression, with many important scientific findings supporting either a correlation or no impact [7,8]. Even though some generalizations may be made regarding cancer, it is important to realize that the interplay of sleep and cancer is likely specific to the tissue and molecular subtype of cancer based on the underlying molecular and physiological processes of each cancer type. Although this makes generalizations difficult, the overarching conclusion is that cancer may have unique interactions with sleep. In understanding the impact of sleep on cancer, it is also important to acknowledge that sleep is strongly intertwined with additional processes that have been independently studied for their effect on cancer, such as circadian rhythm and melatonin secretion. While sleep and circadian rhythms are heavily linked, they are two separate processes. This article will touch briefly on some aspects of the circadian rhythm; however, we will mostly focus on the interaction of sleep and cancer.

Sleep offers an opportunity to broaden our understanding of cancer in the light of understanding the influence of both the states of wake and sleep on normal and aberrant cellular and organ function. Understanding sleep also promotes a better understanding of the influence of these states on the treatment of cancer and the interaction on the body as a whole. In addition to illuminating vulnerabilities, studying sleep offers the opportunity for new paradigms in therapy and improvements in the quality of life for patients with cancer.

## 2. Influence of Sleep on Developing Cancer

### 2.1. Sleep Duration and Cancer Risk

Sleep provides a vital period for several regulatory functions; understanding the impact of sleep duration and disruption on these regulatory functions may enlighten us to the role of sleep duration in the risk of the developing of cancer. Some cancers appear to have a possible relation to sleep duration (Table 1); however, this association is complex and numerous studies addressing this question have reached different conclusions. Longer sleep length may increase the risk for cancer. In a study of the UK Biobank and breast cancer, Richmond found a 1.19 weighted odds ratio in favor of developing breast cancer for each hour of sleep longer than 8 h and a mild protective effect of sleep for less than 7 h [9]. Other studies show an association between longer sleep duration and cancer; for example, there is an association between a sleep duration more than 9 h and a higher risk of colorectal and liver cancer and a sleep duration greater than 10 h and lung cancer [10,11,12]. In contrast, Verksalo et al. described a lower incidence of breast cancer in individuals who had longer sleep duration in a prospective study [13]. Kakizaki also found an increased risk of development of breast cancer in women who slept less than 6 h a day. Two studies studying this same question could not determine a relationship between sleep duration and breast cancer [14,15,16]. Zhou et al. also found that short sleep duration was associated with an increased risk of cancer of the lung, by 16% [17]. Short sleep duration may also increase the risk of colorectal and other cancers [18]. In a long-term follow-up study involving sleep duration, Gapstur et al. found a correlation between shorter sleep duration and the development of prostate cancer [19], and Sigurdardottir et al. also found that sleep disruption and a shorter sleep duration are correlated with an increased risk of the development of prostate cancer in older males [20]. Alternatively, Lu, using a meta-analysis of 10 studies, did not find a statistically significant relationship of short or long sleep duration with an increased overall risk of cancer, but trends were noted for short sleep with the development of prostate cancer, thyroid cancer, and ovarian cancer, and long sleep duration with colorectal cancer [21]. An umbrella review of this topic also supports the conclusion that the impact of sleep duration on carcinogenesis appears to be tissue-specific [20]. This conclusion is also supported by a study by Li et al. which supported both an increased risk of 21% for long sleep duration and colorectal cancer, and a trend for skin cancer with short sleep duration. The fact that the impact of sleep duration appears to be tissue-specific raises questions as to the association being more related to specific underlying physiological pathways intrinsic to each tissue type, or whether the relationship is based on the response of each tissue type to the impact of sleep on more systemic control mechanisms, such as the immune system [22,23]. Evidence from animal data supports a role for sleep restriction in carcinogenesis. One study found that sleep restriction increased polyp development in mice with a genetic predisposition to form intestinal neoplasms [24]. These studies suggest that sleep duration may play a role in the development of some cancers or may exacerbate a pre-existing abnormality, making cancer more likely. These studies also do not give us an understanding of the role that each stage of sleep may play in cancer development. We currently lack any knowledge of the contribution of specific sleep stages (NREM, REM) and perturbations therein as it relates to carcinogenesis.

### 2.2. Sleep Disorders and Cancer Development

Beyond sleep duration, other sleep disturbances and disorders may impact cancer risk (Table 1). Fang et al. used a nationwide, nested case–control study to determine that, through complex mechanisms, sleep disorders may increase cancer risk [30]. Some of this risk may be tissue-specific, but also dependent upon the type of sleep disruption. Zhou et al. found, in a meta-analysis of 11 studies including over 460,000 participants, that sleep disruption increased the overall risk of cancer [17]. Assessing multiple studies, the increased risk of any cancer attributable to sleep disturbances is, at most, a small increase. By exploring this relationship for individual tumor types, we may be able to better understand the impact of sleep disruption on cancer and the mechanism of this relationship. When specifically looking at breast cancer, the hazard ratio increased to 2.38 for women who endorsed insomnia, characterized as non-restorative sleep, difficulty initiating sleep, or difficulty maintaining sleep in a study from Norway of 33,332 women [25]. Yap et al. also found in a meta-review that the presence OSA increased the risk of developing breast cancer by 36% [29].

**Figure 2:**
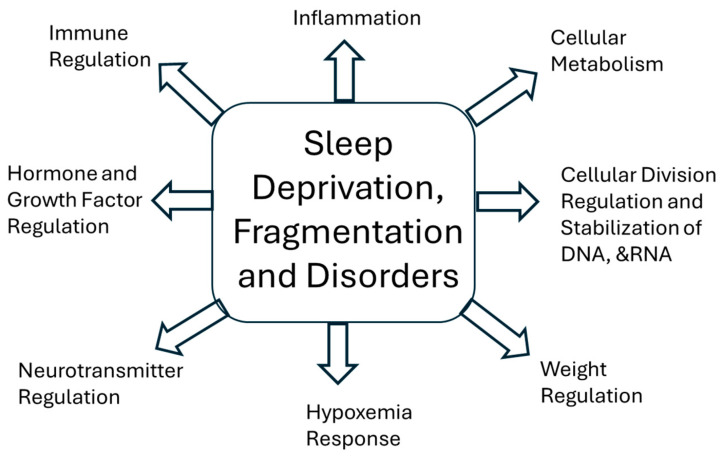
This diagram demonstrates the variety of mechanisms by which sleep, sleep disruption, and sleep disorders may influence cancer development and progression.

Each sleep disorder may influence different molecular processes, and thus the effect of sleep disorders on cancer may be more specific to the type of sleep disruption and tumor type (Table 1). For example, in their meta-analysis, Zhou et al. more specifically found that insomnia was associated with an increased risk of lung cancer with an odds ratio of 1.11, but sleep apnea did not appear to increase the risk of lung cancer [17]. Likewise, Shi et al. performed a meta-analysis with over 570,000 subjects, and found a 24% increase in the risk of cancer in those with the complaint of insomnia, with a higher risk of cancer in women. Shi et al. also found that the highest associated risk for patients with insomnia was developing thyroid cancer [26]. A similar connection was noted by Chui et al., in a study looking at insomnia and breast cancer risk [27]. An increased risk of breast cancer and oral cancer was also described in 43,585 patients with insomnia who used hypnotics [28]. While additional studies have sought to answer this same question, this association was not replicable. For example, an additional study of 110,011 women did not find a link between insomnia and breast cancer [35]. This pattern of conflicting evidence has also been seen for prostate cancer. One Icelandic cohort of 2102 senior men over a 5-year follow-up showed that those endorsing difficulties initiating and maintaining sleep had a 70% higher risk of prostate cancer [20]. Yet, several other studies were unable to confirm this link in prostate cancer. Some of these discrepancies may be based on how insomnia is defined and diagnosed in each study. This potential risk of insomnia and cancer raised questions as to the possibility of the influence of insomnia on hormonal regulation, melatonin, and the immune system (Figure 2). Insomnia in patients with coexisting depression, are known to have elevated the thyroid-stimulating hormone (TSH), as sleep is noted to normally suppress this hormone [36]. Therefore, the increase in TSH could increase the incidence of thyroid cancer in some individuals. Melatonin also decreases in some insomnia patients, and is noted to have an impact on checking cell proliferation, slowing mitotic processes, and influencing the immune mechanisms. Insomnia itself has been linked to changes in inflammation and immune function, which are all plausible pathways by which insomnia may increase the likelihood of the development of cancer [37].

Other types of sleep disorders may increase the risk of some cancers. In addition to individuals with insomnia, Fang et al. found that patients with parasomnias have an increased risk of breast cancer and a significant increase in risk of oral cancers [30]. Fang et al. also described that subjects with obstructive sleep apnea (OSA) had a higher risk of breast, prostate, and nasal cancer. Using OSA as the model for sleep disruption, Gozal et al. found hazard ratios ranging from 1.07 to 1.41 in several types of cancer in individuals with OSA [31]. Although the overall risk of cancer in individuals with OSA was not found to be elevated, individuals with OSA were noted to have a significantly increased incidence of several types of cancer, including melanoma, bladder, lung, liver, cervix, and kidney. In a study involving approximately 20,000 subjects, Marriott et al found that nocturnal hypoxemia was independently associated with cancer, and OSA severity was associated with increased incidence of cancer, but the latter was felt to be related to other coexisting risks [38]. Thus, they concluded that OSA was not an independent risk factor for cancer. Sillah et al. described an association between OSA and an increased risk of breast cancer, uterine cancer, melanoma, and renal cancer based on a population of 34,402 participants [32]. In a study of 163 participants, the incidence of colorectal neoplasia on colonoscopy was three times higher in participants who had polysomnogram verified OSA than participants who did not have sleep disordered breathing [32,33]. Sleep apnea has been postulated to have several pathophysiological cascades, triggering a multitude of mechanisms that could lead to possible increases in the incidence of cancer (Figure 2). The expression of the hypoxia-inducible factor is altered by OSA [39]. The hypoxia-inducible factor regulates processes that help protect cells in the setting of prolonged hypoxemia, but also impact cell cycle and growth. The relatively rapid cycle of hypoxia and recovery clearly influences the cell metabolism, inflammatory and immune response [40]. Similarly, the oxidative stress of sleep apnea may induce changes in DNA and RNA stability [41]. The link of sleep apnea and cancer is very complex and many confounding variables exist, such as obesity [42]. These studies, however, suggest that the potential link between sleep and cancer may depend upon the specific pathways that are interrupted, and that sleep itself may have an impact on stabilizing cell growth and inhibiting carcinogenesis. Therefore, understanding the link between sleep disruption and downstream secondary effects, resulting in mechanistic and microenvironmental changes, is key to understanding the impact of sleep disorders on the development and progression of cancer.

Hypersomnia disorders may also increase the risk for cancer. Higher incidences of head and neck and gastric tumors were found in women with narcolepsy [34]. The mechanisms proposed that could underly the impact of narcolepsy on cancer risk are related to the immune response or the loss of orexin [34]. Narcolepsy with cataplexy (narcolepsy type 1) results from the loss of neurons that produce orexin. Orexin is relevant to narcolepsy as it appears to help stabilize the wake state. However, orexin also has proposed systemic roles, notably including that it may regulate apoptosis through the Orexin1R receptor. This receptor has been found to be expressed in some colorectal and pancreatic tumors, indicating a potential vulnerability of these tumors to orexin [43]. When tumors are subjected to orexin A in vivo or in vitro, there is a significant reduction in tumor size [44]. Interestingly, a dual orexin receptor antagonist, almorexant, with a similar ability to orexin A, also causes apoptosis in pancreatic cancer cells, raising interesting implications for therapy [45]. Thus, it is conceivable that the loss of orexin in narcolepsy could prevent the inhibition of aberrant cell growth. This connection supports the possibility that some forms of sleep disorders, such as narcolepsy, may give additional clues to specific tissue regulation.

These studies suggest that individuals with insomnia, hypersomnia, parasomnia, or OSA may have a higher risk of developing cancer. The link of sleep disruption to cancer may be confounded by changes in immune response, impairment of melatonin release, or changes in weight, and these changes in immune response, weight, and other regulatory factors may make specific types of cancer more likely to occur (Figure 2). Yet it is unclear if these causes of sleep disruption may have a causal link or may be a result of confounding factors that also increase the risk of developing cancer. Thus, the role of sleep duration, fragmentation, and other sleep disorders in cancer development and progression needs further delineation to provide a clearer linkage of sleep disorders and cancer development.

## 3. Sleep Disorders in Cancer

### 3.1. Sleep Disorders in Individuals with Cancer

Many people suffer from sleep complaints, and this rate increases in cancer patients [46]. Sleep complaints are reported by as high as 75% of cancer patients in a variety of studies [47,48] (Table 2). Sixty-six patients with cancer reported a sleep disturbance in a meta-analysis of 160 studies [49]. Although there is no consensus on the term sleep quality, many investigators use different sleep questionnaires, such as the Pittsburgh Sleep Quality Index, to assess overall sleep issues. Performing a meta-analysis of 59 epidemiological studies with different sleep quality questionnaires, Chen et al. found a global prevalence of poor sleep quality in 57.4% of patients with cancer [50]. Both adult and pediatric patients with cancer can have a wide array of sleep issues, which are including but not limited to excessive sleepiness, difficulty initiating or maintaining sleep, and unusual nocturnal movements, similar to the general population [51,52]. The specific type of cancer, the treatment plan, and the patient’s overall course appears to impact the rate of sleep issues [53]. Over three-fourths of non-small cell lung cancer patients complain of poor sleep, most having their sleep symptoms predate the start of therapy [54]. As discussed in the section on the association of sleep disorders and the risk of developing cancer, sleep issues may start prior to the diagnosis and treatment of cancer, and may last years after the individual’s cancer is in remission. This is frequently seen in breast cancer patients, many of whom have sleep issues prior to their cancer diagnosis that are frequently aggravated with chemotherapy [55]. The presence of sleep issues may also increase the likelihood of side effects from the cancer treatment, as Jung et al. found with higher incidence of chemotherapy-induced gastrointestinal symptoms in breast cancer patients with sleep issues [56]. Similarly, patients with head and neck cancers and OSA may have exacerbation of their OSA with therapies such as radiation [57]. In a single-center review of over nine thousand patients, Page et al. found that one in five patients with cancer developed insomnia during treatment [58]. Part of the increase in the prevalence of sleep complaints may be from pre-existing conditions or vulnerabilities, the effects of the tumor on sleep, the influence of cancer-directed therapies, and the stress and emotional burden of a cancer diagnosis. Patients may also have changes in weight, diet, and activity levels that influence sleep and waking. Yet, sleep disruption may also be a prognostic factor in cancer. Palesh et al. found that cancer patients with better sleep efficiency and duration, measured by actigraphy, had lower mortality [59].

### 3.2. Sleep Disorders and Cancer Progression

Interest in sleep and cancer has increased due to increased understanding of how sleep may directly influence cancer, as well as how sleep may regulate mechanisms that may regulate tumor growth and metastasis. As mentioned in the section on sleep disorders in patients with cancer, cancer patients experience high rates of sleep disturbances. These sleep disturbances may, in turn, have profound consequences. In addition to the reduced quality of life, cancer patients with sleep issues have higher rates of mood disorders, shorter time to progression, and reduced survival [60].

In the Wisconsin Sleep Cohort study, with 22 years of follow-up data, subjects with sleep-disordered breathing were found to have a higher mortality rate from cancer than those without sleep-disordered breathing [61]. This group also found that individuals with increased severity of hypoxemia had higher cancer-related mortality rates. Huang et al. also found a higher mortality rate among individuals with advanced lung cancer and sleep apnea [62]. Similarly, an increase in mortality in patients with melanoma and sleep apnea was noted in a five-year follow-up study [63]. The link between sleep issues and cancer-related mortality is likely complex as, contrary to these findings, the Women’s Health Initiative population study failed to find a link of insomnia with cancer mortality [64].

In determining the impact of sleep disorders on cancer progression, it is important to look beyond mortality rate. Sleep issues may impact other aspects of cancer progression, as well as recovery. For example, OSA has been associated with an increase in metastasis rates [31]. Untreated OSA results in intermittent hypoxia, which in turn can give rise to more aggressive growth in melanoma. This has been replicated in animal studies with experimentally created intermittent hypoxia [65]. Martina Garcia et al. found a direct relationship between an increase in the severity of sleep apnea and an increase in the aggressiveness of melanoma [66,67]. Despite this, the same group did not find an increase in markers for aggressiveness in breast cancer in patients with concurrent OSA, suggesting that there may be a tumor-specific component to this interplay [68]. Other sleep disorders such as insomnia may also impact cancer survival. Wang et al. found that ovarian cancer patients with insomnia had shorter survival times [69]. Similarly, a meta-analysis of studies of sleep and multiple cancer types conducted by Strom et al. found that disturbed sleep during treatment was associated with shorter time to disease progression and lower survival [70]. In animal studies, mice that had chronically fragmented sleep had accelerated tumor growth [71]. This raises the question of what are the underlying mechanisms that regulate the connection between sleep disruption or intermittent hypoxemia on increasing tumor growth. It is most likely that both may have a subtle effect through multiple pathways. The reason for this increase in tumor growth and aggressiveness in sleep fragmentation may be connected to the hormonal, immune, and cellular metabolic factors noted above; however, other factors may also be involved in accelerating tumor growth. Beyond the effect of hypoxemia, sleep disruption itself impacts carcinogenesis and cancer growth through its impact on angiogenesis, immune response, metabolism, and hormone regulation (Figure 2). Understanding the mechanisms underpinning the relationship between sleep and cancer has allowed researchers to better define questions, and has led to increased interest in the field. For example, there is a growing body of work on sleep, the immune system, and cancer. There is a bidirectional relationship of the immune system and sleep, with both processes regulating the other. This is demonstrated in the increase in sleep complaints with increased levels of inflammation frequently seen in cancer patients [72]. Increased inflammation, such as that seen in cancer, can have a key impact on sleep quality, fatigue, pain, and mood [73]. Cancer progression may be impacted by the inflammation caused by sleep issues [74]. Fragmentation of sleep is associated with increased inflammatory markers and glucocorticoid and catecholamine levels. Elevated glucocorticoid and catecholamine levels may weaken the immune response and promote angiogenesis. Beyond inflammation, sleep disruption has been shown to impact the expression of other growth factors and hormones, as well as their receptors. Through this and other mechanisms, sleep disruption and fragmentation aid tumor progression. Beyond the direct impact on cancer, sleep disruption can negatively impact healing and resilience by limiting cognitive and emotional spheres. This prolongs the negative impacts of cancer, even after there is no evidence of disease.

Sleep disruption and fragmentation in cancer patients result in multiple physiological changes that impact tumor progression and recovery in a tumor-specific manor. Understanding this relationship may help us to better care for cancer patients; therefore, more research on this topic is needed [48].

## 4. The Impact of Cancer and Cancer Treatments on Sleep

Cancer and cancer-directed therapies may have direct and indirect effects on the brain circuits involved in the regulation of the wake and sleep states. These effects may be altering the molecular signaling pathways of the homeostatic and circadian drives. These signaling pathways may be influenced by changes in cellular metabolic products, hormonal regulation, and inflammatory and immune products. Similarly, the side effects of chemotherapy, radiation therapy, and surgery can include nausea, pain, and fatigue, which frequently result in disturbed sleep. Sleep changes can also occur because of the changes in activity level, diet, and mood that come with cancer and cancer treatment. The understandable emotional toll of the diagnosis of cancer creates substantial stress on the individual, worsening their quality of sleep.

In understanding the influence of cancer on sleep and sleep issues, it is important to understand the impact on each stage. Unfortunately, very few studies have used polysomnography to characterize sleep stages in cancer patients. One study that explored this question was conducted by Parker et al., who used ambulatory polysomnography to characterize sleep stages in 114 cancer patients, and found that people with cancer have reduced total sleep and less slow wave and REM sleep compared to people who did not have cancer. In this study, cancer patients also had increased arousals and more sleep continuity compared to the participants who did not have cancer [75]. This study also found that individuals who slept more during the day had less sleep at night. Reinsel et al. conducted a two-night sleep study protocol of patients with breast cancer, and found a normal average sleep efficiency at 86.7%, with relatively normal distribution of sleep stages. Patients with a higher intensity of sleep disruption had higher periodic limb movements indices in sleep [76]. Unfortunately, the subjects were not asked about symptoms of restless legs syndrome. Although the connection between periodic limb movements in sleep and breast cancer is unclear, studies like these suggest that each cancer type may have a unique effect on sleep regulation.

Physiologically, cancer may also play a direct role in sleep regulation and drive. Frequently, tumor–immune system interactions result in the release of several cytokines that contribute to the homeostatic sleep drive, including TNF-α, I-1β, and IL-6, which can cause daytime fatigue and lead to sleep problems such as insomnia and sleep disruption [77]. These cytokines and molecules can increase after cytolytic therapies. The hypothalamic sleep–wake circuitry may also be impacted by cancer through the impact of cancer on hormones such as leptin and ghrelin [78]. More directly, cancer may directly invade the brain centers that control sleep–wake state. Craniopharyngiomas specifically invade the hypothalamus, potentially disrupting both centers in the control of sleep and circadian rhythm [79]. Patients with damage to the hypothalamic center may show an inability to stabilize their sleep state, including REM sleep, similar to the instability of sleep state that occurs in narcolepsy. The diagnosis of cancer and the implications of cancer can influence the patient’s emotional balance. This secondary effect on mood and mood disorders, such as depression and anxiety, can frequently present sleep issues [80]. Lastly, patients with cancer may change their daily routine, causing disruption to their schedule and causing less activity, which subsequently influences sleep. Thus, cancer has numerous pathways to cause disruption of sleep.

In addition to the physiological and psychological burden of cancer, the treatment of cancer may also disrupt sleep. Several studies have demonstrated that both radiation therapy and chemotherapy cause fatigue and insomnia [81]. Medications such as glucocorticoid steroids are commonly used in cancer therapy regimens, yet these frequently cause insomnia, especially when the steroid has a longer half-life or is taken later in the day or requires twice-daily dosing [82]. Other chemotherapies are also associated with insomnia. Kiss, in a meta-analysis, found insomnia was a common side effect for most chemotherapies, hormonal therapies, and immunotherapies [83]. This included finding that checkpoint inhibitor therapies had similar rates of insomnia as other immuno- and non-immuno-targeted therapies, with odds ratios for insomnia ranging from 1.4 to 1.49 [83]. In another study, Ancoli-Israel et al. found that in breast cancer patients, chemotherapy was shown to increase the severity and chronicity of insomnia, especially in those who had a tendency for insomnia prior to the cancer diagnosis [55]. Immunotherapies for cancer may also influence sleep and the indirect pathways that impact sleep [84]. Zarogoulidis et al. studied 49 patients with lung cancer undergoing only immunotherapy directed toward programmed cell death ligand 1 (PD-L1), and found that those with higher PD-L1 expression had a higher likelihood of improvement in sleep latency and sleep duration reduction in fatigue compared to patients with lower PD-L1 expression [85]. They also found that after nine months of therapy, those who had a complete response of their tumor also had near-complete resolution of their sleep issues. This included cancer-related fatigue, as well as fatigue related to confounding factors. Patients who had partial and complete response of their tumor also had an improvement in emotional status, pain status, fatigue, and overall sleep quality. There is a growing interest in modifiable moderating factors of the relationship between cancer and sleep, such as the gut microbiome. Studying fatigue in cancer patients, Hajjar et al. found that the gut microbial species, Eubacterium hallii, was associated with lower fatigue scores, whereas Cosenzaea was associated with high fatigue scores [86]. This demonstrates the inter-relationship of multiple systems that may change with cancer and cancer therapies, and their potential influence on sleep.

Overall, the impact of cancer and cancer treatment on sleep may occur through both tumor-specific and general systemic mechanisms. Additional studies are needed to better define both these direct and indirect pathways, and to determine the best interventions for patients.

## 5. The Effects of Treating Sleep Disorders in Cancer Patients

In light of the possible impact of sleep disorders on carcinogenesis, as described above, treating a cancer patient’s sleep disorders could conceivably influence their outcomes. To date, data are still limited on the impact of treating sleep disorders on cancer outcomes. Most studies show that treatment of the sleep disorder improves sleep-related symptoms. As an example, Ganjei et al. found that treatment of OSA with continuous positive airway pressure improved the daytime sleepiness and fatigue in patients with cancer and OSA, but the study did not include any data on the subsequent cancer status [87]. Similarly, the treatment of insomnia in patients with cancer with pharmacological and nonpharmacological therapies improved the insomnia symptoms, with little information on the effect on cancer progression [88,89].

Although intermittent hypoxia triggered more aggressive growth of melanoma and other tumors in both animal and human studies, clinical evidence of improved cancer-related outcomes in those treated for their sleep issues is sparse [65,66]. In a small study of patients with sleep apnea, Gharib et al. found a reduction in cancer-associated transcriptional signatures with continuous positive airway pressure (CPAP) treatment of OSA, suggesting that an improvement in sleep hindered the environment for tumor growth [90]. Another study found a decrease in inflammatory factors, improved immune response, and reduced vascular endothelial growth factor in patients who were complaint with long-term CPAP [91]. Other treatment paradigms for sleep disorders may add to other improvements in cancer outcomes [92]. Insomnia can be treated with cognitive behavioral therapies for insomnia (CBT-I). For cancer patients with insomnia, CBT-I can improve not only their sleep quality, but also their quality of life, whilst also decreasing comorbidities, and potentially even reducing healthcare costs [93,94,95]. CBT has been shown to improve insomnia, in fact, Peoples et al. found that CBT resulted in better outcomes than CBT in combination with armodafinil [96]. In addition to CBT-I, other nonpharmacological interventions for insomnia may provide some benefits to cancer patients [97]. These include acupuncture and progressive muscular relaxation [98,99,100]. Medications appear to have mixed results. Few pharmacological studies examining the treatment of insomnia exist. In one small study of temazepam versus a melatonin group and a placebo group, both melatonin and temazepam showed an improvement in sleep issues, but no improvement in quality of life [101]. Another small study of 30 patients with advanced cancer demonstrated that trazadone as a treatment for insomnia both improved sleep quality and reduced nightmares [102]. However, a meta-analysis of seven randomized studies with trazadone in the general population failed to show significant improvements in sleep latency, duration, or efficiency. Despite this, patients randomized to trazadone did perceive better sleep quality in three of the studies [103]. Melatonin at a nightly dosage of 3 mg was also found to reduce insomnia severity in cancer patients [104]. Exogenous melatonin at low doses (1–3 mg), timed appropriately, can also have an effect on circadian rhythm, but at high doses, it may provide antioxidant properties. In another study of patients with breast cancer and neuropathy, pregabalin improved both insomnia and pain better than duloxetine [105]. The key information lacking for these studies is the impact on the cancer itself. Further research is needed to elucidate the benefits of treating sleep disorders for cancer patients.

## 6. Clinical Approach to Sleep Issues in Patients with Cancer

The approach to sleep issues in patients with cancer should be holistic, including a review of daily routine, sleep and wake timing, sleep environment, diet, supplements, additional medical issues, recent therapies, and attitudes towards sleep. Sleep issues in cancer patients can fall into three subtypes: sleep issues independent from the cancer, sleep issues related to the cancer or cancer therapy, or sleep issues related to the stress of the diagnosis. Understanding the driver is important for the clinician to best address the sleep issue. Each of these possibilities are not exclusive, and thus deserves clinical introspection as the drivers may offer unique opportunities in approach. In many situations, treating the sleep issue as well as the underlying driver may result in better outcomes.

As many as three-fourths of patients with cancer may have a sleep-related complaint. A methodical approach should be taken when evaluating sleep disturbances in cancer patients (Table 2). Simple screening questions may aid the clinician to identify a sleep issue. The following three questions from the Patient-Reported Outcomes Measurement Information System (PROMIS) questionnaire may provide an entry route: “Are you sleepy during the day”, “Do you feel refreshed during the day”, and “Are you satisfied with your sleep” [106]. The backbone of the evaluation rests on the patient’s history. For clinicians who are not formally trained in sleep medicine, questionnaires such as the Pittsburgh Sleep Questionnaire Index can provide general coverage of sleep issues, with subsections highlighting specific symptoms of sleep apnea, insomnia, or movement issues (Table 3) [107].

A clinical history should detail the major characteristics relating to possible sleep disturbances, their time of onset, and their course over time in relationship to the patient’s cancer. The cancer type may also play a role, as well as the therapies and treatment course. To appropriately support their patient, the clinician should ensure that they have a detailed understanding of the patient’s sleep complaints, as well as the factors that may be propagating the sleep issues. Understanding the patient’s daily schedule and habits on both work and non-work days necessitates tracking daily therapy routines, sleep location, and environment, habits to prepare for sleep or during middle of the night when awake, use of other substances (caffeine, alcohol, herbs, recreational drugs, etc.), and a description, if possible, from their bed partner of movements, snoring, or witnessed pauses in breathing. Clinicians should gather clues to typical work schedule, wake-up time without an alarm, mealtime, and activity times through the day to assess the timing and strength of the patient’s circadian rhythm (Table 4).

Approximately one-fifth of workers work a rotating or night shift; thus, shift work may be an important factor for sleep deprivation and circadian rhythm. Furthermore, understanding the patient’s perspective on sleep and their daily symptoms is important to assess if the patient views sleep as an important or not-so-important aspect of their health. Additionally, understanding the treatment schedule may also help, as these can also attenuate or induce brief shifts in sleep–wake times. Given the limited availability of some treatments, some patients may need to arrive at the health facility very early, earlier than their normal start of day, or stay late at night to receive their therapy, thus causing added disruption to their schedule. Tools such as a sleep diary or actigraphy can help elucidate the patients sleep–wake pattern, and provide evidence for a circadian rhythm issue.

**Figure 3:**
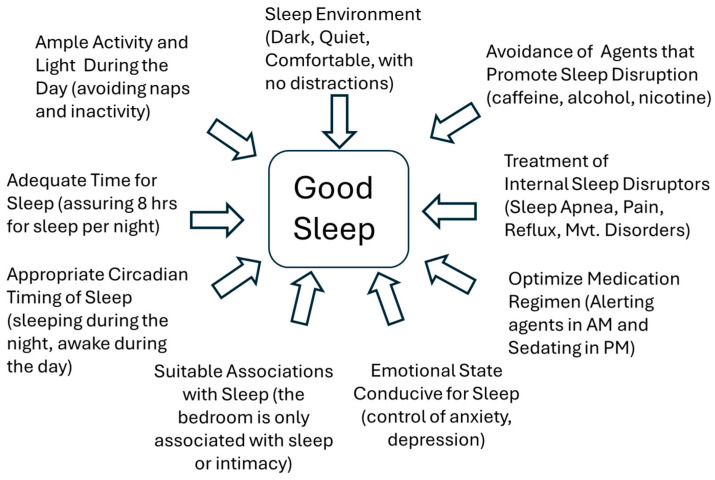
Many factors can influence sleep. This diagram illustrates the variety of components involved in achieving good sleep and the domains by which sleep may be disrupted.

For many patients with cancer, multiple drivers may play a role in the development and accentuation of sleep disturbances, and conversely, their importance for good sleep (Figure 3, Table 5). Thinking of the homeostatic drive, factors influencing adenosine, cytokines, or other metabolic byproducts may be either enhanced or blocked. Caffeine, which blocks the effects of adenosine, may play a significant role in insomnia and sleep disruption, leading to more daytime fatigue, and thus added caffeine use.

Other factors such as the total time in bed; the timing of sleep; the possibility of something disturbing sleep(such as OSA, or periodic limb movements), or reflux; or external factors, such as an uncomfortable bed or a noisy or lighted sleep environment, should be considered. Also, mood and conditioned associations, such as worrying in bed, may contribute to poor sleep. The clinician should consider agents that may initiate these factors, as well as additional drivers that may perpetuate sleep issues (Table 5). Patients may start with insomnia as part of chemotherapy, but perpetuate this insomnia with maladaptive behaviors, such as sleeping with the television on. Some patients may require formal sleep testing, such as polysomnography, especially when considering that the patient may have a sleep-related breathing disorder, nocturnal movement disorder, or is at risk for injury from nocturnal events (Table 6).

Further investigations should address specific questions assessing symptoms of OSA, restless legs syndrome (RLS), insomnia, and hypersomnia (i.e., narcolepsy). Additionally, the subjective estimate of daytime sleepiness may provide further clues [108]. The Epworth Sleepiness Scale (ESS) is a reliable measure to assess subjective sleepiness, but it has not been specifically validated for cancer patients [108] (Table 3). Clinically significant daytime sleepiness is demonstrated by a score of greater than or equal to 10. Originally validated in patients with OSA, sleepiness scores from 10 to 15 are typically associated with OSA, whereas lower scores are more likely in those with insomnia or restless legs syndrome, which are typically low; thus, higher scores raise suspicion of primary hypersomnia disorders or additional causes of sleepiness.

### 6.1. Clinical Approach to OSA

Obstructive sleep apnea can be prevalent in patients with cancer ranging from 4 to 50%, and some outcome studies suggest that certain cancers progress in relation to the severity of the OSA [62,109]. Consequently, given the prevalence in and potential impacts of OSA on cancer patients, it is important for clinicians to screen for sleep apnea [40]. Symptoms to monitor include daytime sleepiness, snoring, or witnessed apnea events by bedpartner. To best identify patients at risk for sleep apnea, clinicians can use the STOPBANG questionnaire. The tool contains eight items, and a score of three indicates that a patient should be further evaluate for sleep apnea (Table 3) [110]. While the STOPBANG questionnaire was validated in the general population and not specifically for cancer patients, Wong et al. determined that it had a 73% sensitivity and 71% specificity in a group of 249 cancer patients [111]. In the laboratory, polysomnography is the gold standard for determining the type and severity of sleep-related breathing disorders (Table 6). Treatment plans are typically based upon these polysomnographic results and adapted to the severity and patient’s acceptance of the therapy. To determine severity, the apnea hypopnea index is typically used to indicate the average number of respiratory events per hour of sleep [112]. For adults, 0–5 is considered normal, while 5–15 is mild, 15–30 is moderate, and >30 events per hour is severe. Patients without daytime symptoms and mild sleep apnea may respond to conservative therapies such as nasal steroids or decongestants, sleeping in the side position, weight loss, and avoiding alcohol and respiratory suppressants. If there is a presence of daytime symptoms or patients have moderately severe sleep apnea, there is a possible benefit to the use of oral appliances, positive airway pressure devices, or surgery. Notably, no matter the degree of sleep apnea, all severity levels may respond to positive airway pressure. Special consideration may be given to challenging issues. Those patients with mucosal ulcers due to chemotherapeutic agents, or radiation therapy or skin breakdown from radiation or surgery, may be physically limited for wearing a CPAP mask or oral appliance. Patients who have developed claustrophobia or significant anxiety may benefit from less intrusive CPAP interfaces such as nasal pillows, and patients may find less leaks from the mask using a variety of “CPAP” fitting pillows. Finding a mask that is comfortable and well-fitting is crucial to success. Nevertheless, the patient must accept and see the therapy as a necessary and key part of the overall strategy to improve their prognosis and health.

### 6.2. Clinical Approach to RLS

Restless legs syndrome (RLS) can be easily diagnosed by the constellation of having uncomfortable and unpleasant sensations with the urge to move their legs that is worse with rest, totally or partially relieved with movement, and primarily occurring in the evening or night. Although the overall prevalence of RLS in patients with cancer is not known, few case reports suggest RLS can be observed. Moreover, some therapies, such as dopamine antagonists used to treat nausea and aromatase inhibitors used in breast cancer, may increase the frequency of RLS [113]. RLS also may become prevalent in cancer patients as sequelae of cancer treatments, such as anemia, iron deficiency, the use of antipsychotics for sleep, and even environmental issues, such as being still for prolonged periods of time in small spaces, are risk factors for exacerbating RLS. In a small study, Yennurajalingam et al. found that 38% of patients with advanced cancer had symptoms of RLS [114]. Conversely, adjunctive therapies used to treat cancer and cancer treatment-associated pain and nausea, such as gabapentin and narcotics for pain, or pregabalin and benzodiazepines, may improve RLS symptoms [115]. RLS frequently causes sleep disruption and reduces overall total sleep time, triggering difficulty falling asleep and increasing daytime fatigue, consequently adding to the comorbidity of cancer. Gabapentinoid medications can provide significant improvement in RLS symptoms and improve sleep. These medications can be used as first-line therapies for RLS, and are an excellent option for treating RLS symptoms in patients with cancer. Focusing on alleviating the patient’s symptoms of feeling an uncontrollable urge to move is key, especially when their therapy requires them to remain still for prolonged periods of time. Similarly, treatment using oral iron or iron infusion or therapy of low iron levels, ferritin levels below 75 mcg/L, or iron saturation below 30% may reduce symptoms and medication requirements [116,117].

### 6.3. Clinical Approach to Excessive Sleepiness

Frequently, patients with cancer complain of excessive sleepiness. This is frequently confused with fatigue, the sense of loss of energy. The distinguishing feature is the ability to fall asleep, as fatigue is the loss of energy without an increased ability to fall asleep, and sleepiness is an increased ability to sleep or doze. Common drivers of sleepiness include sleep deprivation, sleep disruption such as sleep apnea, or factors adding to the homeostatic pathway, such as adenosines or cytokines, medication effects, or circadian rhythm issues (Table 3). Some of these patients should be considered for polysomnographic evaluation, especially if there is snoring or concerns of hypoventilation (Table 6). Less commonly, if these etiologies are excluded, the clinician should consider the possibility of a primary hypersomnia issue, such as narcolepsy or idiopathic hypersomnia. These conditions require further evaluation and treatment in a sleep center. Narcolepsy is divided into type 1, associated with loss of the wake-stabilizing neurotransmitter orexin (or also defined by presence of cataplexy), and type 2, which is not associated with cataplexy. Patients with narcolepsy may have symptoms related to the dysregulation of REM sleep, including irresistible bouts of excessive daytime sleep, hypnogogic and/or hypnopompic hallucinations, or sleep paralysis. The diagnosis of narcolepsy is confirmed by an overnight monitoring of sleep of least 6 h, followed by a multiple sleep latency test (MSLT) demonstrating a mean sleep latency of less than 8 min and the presence of two of the five naps showing sleep-onset REM periods [112]. Other primary hypersomnias, such as idiopathic hypersomnia, may be possible. Idiopathic hypersomnia is defined as a continuous feeling of dogged sleepiness despite prolonged periods of sleep, similar to that described by some patients undergoing radiation or chemotherapy. Idiopathic hypersomnia is uncommon; however, it is important to consider the differences. The mainstay of therapies for primary hypersomnias is treatment with wake-promoting agents and stimulants during the day and therapies to consolidate and deepen sleep at night. Newer stimulants, such as pitolisant and solriamfetol, add to the existing list of stimulants, such as modafinil and armodafinil. Medications such as sodium oxybate and low-sodium oxybates are proving helpful to improve daytime sleepiness in both narcolepsy and idiopathic hypersomnia [118]. While a mechanistic relationship between narcolepsy, hypersomnia, and cancer is still unclear, the clinician should first address issues such as assuring enough time is dedicated to sleep, optimizing medication timing of sedating medications in the evening and alerting drugs in the morning, addressing sleep disruption from sleep apnea, reflux, pain and environmental disrupters, and optimizing the patient’s circadian rhythm by including light exposure and activity during the day.

### 6.4. Clinical Approach to Insomnia

Insomnia may present at any time in the course of a patient’s cancer. Insomnia is defined by difficulty initiating sleep or difficulty maintaining sleep with daytime consequences. In cancer patients, insomnia can be triggered by or worsened by the cancer itself; the medications used to treat the cancer; the anxiety and fear associated with a cancer diagnosis; the use of alcohol, caffeine or herbs; and a lack of activity during the day (Figure 3, Table 4). The clinician should consider the patient’s comorbidities, including underlying anxiety and depression, which are frequently associated with insomnia. Insomnia may be triggered by a sudden change in the patient’s life or even medication, yet insomnia commonly persists as a result of adopting a maladaptive behavior. These behaviors regularly include sleeping with the television or lights on, sleeping in a recliner chair or away from the bedroom, reading on a computer or getting involved in an engaging activity before bed, or having foods such as alcohol or coffee in the evening. A few studies have attempted to measure the effects of specific nonpharmacological approaches to insomnia. Jung et al. found that cognitive behavioral therapy for insomnia (CBT-i) was effective in cancer patients for reducing sleep complaints and decreasing dependence on hypnotics [98]. CBT-I can be directed at restructuring maladaptive beliefs and breaking these sleep-prohibitive behaviors, and may benefit these patients in other domains [119,120]. Acupuncture was also found to reduce symptoms of insomnia in cancer patients [99]. Additionally, cancer patients may turn to supplements and herbs to improve their prognosis or reduce their symptoms, although these should first be cleared by their oncologist to ensure that the herbs and supplements do not interact with their chemotherapy. In one study, melatonin at 3 mg was found to reduce insomnia symptoms, and in another study, lavender combined with footbaths appeared to provide some benefits [101,121]. The clinician may also wish to identify possible over-the-counter remedies or supplements that do not improve sleep long-term, but may interact with other aspects of the patient’s cancer therapy.

Insomnia is best approached with a multi-pronged strategy. Important components of this approach include promoting the correct clues for sleep and waking, bringing an end to maladaptive behaviors, optimizing current medications, and then considering medication (Table 5). Patients should be counseled to set a regular schedule, accentuating activity and light during the day, avoiding napping and caffeine during the day, while making sure the bedroom is dark, comfortable, and quiet at night. These features promote the natural signals for sleep and wake periods. Including CBT-I will also encourage these behaviors, while working to forgo maladaptive behaviors. CBT-I does require trained personnel, and these resources can be limited. Digital CBT-I can provide an accessible effective alternative to in-person CBT-I [122]. CBT-I can be offered as an online or phone application in the form of cost-effective programs that have demonstrated benefits, especially to those patients with limited access [122,123,124]. While few studies exist on many of these programs, the program Somryst (formerly the SHUTi application) has demonstrated effectiveness in the published studies, and is cleared by the United States Food and Drug Administration (FDA) for digital CBT-I. Given that these patients may be on many medications, many cancer patients are willing to try behavioral therapies, yet those with severe insomnia will still request hypnotic medications [125]. Pharmacotherapy for insomnia is best when directed as a short-term therapy, and most helpful for those sleeping less than 6 h due to this group having a limited response to CBT-I [126]. Although frequently prescribed initially as short-term, many hypnotic medications become chronic in this patient population [127]. Medications such as benzodiazepine receptor agonists (zolpidem, eszopiclone), low-dose doxepin, or orexin receptor antagonists (suvorexants, lemborexants, daridorexants) are approved by the FDA for insomnia. Each provide benefits for the general population, but evidence for cancer patients is minimal [92]. These patients have successfully tapered off medication with continued sleep benefits through the use of directed CBT-I [98]. For patients who have intractable insomnia, it is important to consider additional underlying sleep disorders, such as restless leg syndrome or sleep apnea, or other underlying medical or psychiatric disorders. If these are identified, treatment of the underlying issue, in addition to addressing the insomnia, is important to best care for the patient [128].

### 6.5. Clinical Approach to Parasomnias

Parasomnias as a result of cancer is an area with little information. While some patients with cancer may develop nocturnal events, many of these are during treatment, and may involve periods of delirium caused by metabolic derangement, medications, or toxins, and can be confused for a parasomnia (Table 3). These nocturnal events can be difficult to differentiate from classical NREM and REM-related parasomnias, even for a highly skilled clinician. As with other sleep disturbances, a clear description of the events is the cornerstone of the evaluation. Clinical details such as time of occurrence, duration, frequency of events, behavior and clarity of speech, retained memory for the event, dream mentation, and specific characteristics observed, such as eyes open or closed, are clues to the underlying etiology. Additional features such as a family history of events, age of onset, and exacerbating factors are also helpful. If these events appear to increase the risk of injury to self or others, or the patient has additional symptoms of other sleep disorders, a video polysomnography with an extended EEG montage should be performed [129] (Table 6). Typically, parasomnias are divided into the stage of sleep from which they arise [112]. Disorders of arousal which arise from NREM and consist of sleepwalking, sleep terrors, and confusional arousals usually occur in the first third of the night, and comprise variable behaviors lasting typically minutes, for which the patient has minimal or partial memory of the event. Although there are other REM parasomnias, REM sleep behavior disorder is associated with patients acting out their dreams, which many times are violent. These events are more common in the latter half of the night, and patients and bedpartners may be seriously injured. A key feature of all these parasomnias is that the behavior described varies between events. When events do not vary and appear to have the same pattern of movements with each episode, these stereotypical events are more concerning for epileptic seizures. Epileptic seizures are classically stereotypic, demonstrating the same behavior each time, and can vary in onset and duration during the night. These events, too, require a more thorough investigation, as the seizures may be a result of metastasis to the brain.

Cancer patients can have a wide array of sleep issues arising from many factors; thus, taking a personalized approach to treatment is essential. As these cases may have multiple domains of medical issues, the same range of domains may exist driving the sleep symptoms in these, requiring a multifaceted diagnostic and therapeutic approach.

## 7. Future Directions

Sleep influences many physiological and molecular processes. Understanding how these processes impact the development and progression of specific cancer types could help us to better elucidate the pathophysiology of and risk factors for cancer. Further research is needed to understand these mechanisms, as well as the individual impact of each stage of sleep on these processes. These same pathways may present opportunities for therapeutic intervention both directly influencing the cancer, as well as improving the resilience and quality of life of patients with cancer.

## 8. Conclusions

In summary, the relationship between sleep and cancer is complex and confounded by many variables. Additional research is needed to delineate the mechanisms by which various sleep issues impact the mechanisms that underpin cancer risk and development. While the impact of sleep on various cancer types is still not well defined, it is important for clinicians to evaluate for and treat sleep disturbances in cancer patients, as this may improve overall health, quality of life, and possibly improve the individual’s cancer outcomes.

## Figures and Tables

**Table 1:** Cancer types associated with sleep issues.

Sleep Issue	Cancers Found in Association	Sources
Prolonged Sleep Duration	Breast cancer, colorectal cancer, liver cancer, lung cancer	[9,10,11,12]
Shortened Sleep Duration	Breast cancer, lung cancer, colorectal cancer, prostate cancer, skin cancer	[14,17,18,19,20]
Insomnia	Breast cancer, lung cancer, thyroid cancer, oral cancer, prostate cancer	[17,20,25,26,27,28]
Obstructive Sleep Apnea	Breast cancer, prostate cancer, nasal cancer, melanoma, bladder cancer, lung cancer, liver cancer, cervical cancer, kidney cancer, uterine cancer, colorectal cancer	[29,30,31,32,33]
Parasomnias	Breast cancer, oral cancer	[30]
Hypersomnia	Gastric cancer, head and neck cancer	[34]

**Table 2:** Common sleep complaints in patients with cancer.

Sleep Complaint	Behaviors	Disruptors	Disorders
Insomnia	Review time in bed (association) and timing of sleep (circadian issue), napping, activity Inquire into the daily routine and sleep-associated behaviors	Inquire into sleep environment Medications, supplements, caffeine, ETOH, nicotine	Sleep-related breathing disorders, pain, reflux, other medical, and psychiatric issues
Excessive Sleepiness	Review time in bed dedicated to sleep and timing of sleep (circadian issue) Inquire into daily routine and sleep-associated behaviors	Inquire into sleep environment Medications, supplements, caffeine, ETOH, nicotine	Sleep-related breathing disorders, pain, reflux, other medical, and psychiatric issues
Sleep-Related Breathing Issues	Ask about sleep position, ETOH, nicotine airway irritants	Weight changes, reflux, nasal congestion	Sleep-related breathing disorders, lung, heart, brain, neuromuscular disorders
Restless Legs and Other Nocturnal Movement Disorders	Ask about environment the movement occurs in, timing of movement and relationship to caffeine, ETOH, nicotine and stress	Caffeine, ETOH, diet	Restless legs syndrome, periodic limb movement disorder, other sleep transition movements, and neurological issues
Nocturnal Events—Parasomnias, Nocturnal Seizures		Other sleep disorders, ETOH, short-acting hypnotics	Parasomnia, nocturnal seizures, delirium

**Table 3:** Sleep questionnaires by category.

Questionnaire Category	Questionnaire	Questionnaire Overview
Introductory Questions	PROMIS Sleep Questions	“Are you sleepy during the day”, “Do you feel refreshed during the day”, and “Are you satisfied with your sleep”
General Sleep Assessments	Pittsburgh Sleep Quality Index	General questionnaire of 19 items for review of sleep issues
	Sleep Disorder Questionnaire	175-item assessment of sleep habits and disturbance
	Sleep Diary	To track sleep schedule and time
Daytime Sleepiness	Epworth Sleepiness Scale	Able to quantify subjective sleepiness over time
OSA	STOP-BANG Questionnaire	Assesses risk of underlying obstructive sleep apnea
Insomnia	Pittsburgh Sleep Quality Index	Can also be used to track sleep symptoms over time
	Insomnia Severity Index	Assesses severity and impact of insomnia on the patient
	Athens Insomnia Scale	Assesses severity of insomnia
RLS	International Restless Legs Syndrome Scale	Questionnaire to assess the impact of RLS on the patient—can be followed over time

**Table 4:** Evaluation approach for sleep complaints in patients with cancer.

Clinical Approach to Evaluating Sleep Complaints in Patients with Cancer
Sleep History Bedtime routine and schedule (bedtime and waketime) for work and nonwork days Sleeping environment (specifically presence of stimulating items, amount of light, noise, temperature, and bed comfort) Disruptors of sleep Internal—pain, nausea, reflux, breathing issues External—light, noise, uncomfortable environment Personal history of sleep disorders Personal history of sleep disorder symptoms (daytime fatigue, morning headache) Personal history of maladaptive behaviors (such as needing to perform specific stimulating routines before bed, worrying or reviewing a mental list in bed, sleeping on couch, or leaving lights or television on at night) Beliefs about sleep or features that help or impair sleep History from bed partner (snoring, witnessed apneas, abnormal movements) Presence of insomnia or hypersomnia (Epworth Sleepiness Scale) Presence of nocturnal events Daily activities Daily routine including meals, activities, social interaction, and sunshine exposure Work history and job activities and environment Intake amount and timing of alcohol, caffeine, and/or supplements Cancer History Treatment regimen Therapy dosage, timing, and adverse effects Relationship of symptoms to the treatment Additional medical or mental health concerns Review medication list Current or personal history of substance use

**Table 5:** Approach to good sleep.

Sleep Environment
Dark, quiet, comfortable bed and pillow; temperature (68–72 F°)
**Adequate time for sleep**
Dedicating 8 h for sleep
**Circadian Rhythm Timing and Reinforcement**
Activity, exercise, sunlight, regular meal times, social interaction during the day
Decreased light (especially blue light blocking), activity, large meals, and social interaction in evening close to bedtime
Consider a hot bath or shower in the evening
**Promoting the Homeostatic Drive**
Activity for brain and body, avoidance of caffeine
**Encouraging Good Sleep Associations**
Bedroom is only for sleep or intimacy, no television or cell phone, no exercising, working, fighting, worrying in bedroom
Possible use of worry book in a room other than bedroom
**Avoidance of Disrupting Agents**
Avoid caffeine, alcohol, and nicotine products
**Optimizing Medication Regimen**
Maximizing alerting agents in the AM
Timing sedating agents in the PM

**Table 6:** Indications for polysomnography,.

Polysomnography is routinely indicated for:
Evaluation for sleep apnea Differentiating central vs. obstructive sleep apnea Evaluation for sleep-related hypoxemia Evaluation for hypoventilation Evaluation for narcolepsy/primary hypersomnolence in combination with multiple sleep latency tests the next day Positive airway pressure titration for patients requiring nocturnal respiratory support Evaluation prior to upper airway surgery when surgery is being considered for snoring or OSA Patients with heart failure or cardiovascular disease who have disturbed sleep, nocturnal dyspnea, snoring, or other nocturnal symptoms suggestive of sleep-related breathing disorders Patients who have symptoms of sleep apnea or hypoventilation when symptoms persist despite appropriate medical management Evaluation of sleep-related symptoms in patients with neuromuscular disorders Evaluation of patients with nocturnal movements and daytime sleepiness Subsequent sleep studies needed for follow up: Patients with oral appliance treatment for OSA after clinical titration Following appropriate healing from surgical treatment to assess residual OSA Re-evaluate return of symptoms in patients with SRBDs after surgical or dental treatment Re-evaluate PAP settings in patients with SRBD after significant changes in weight Re-evaluate patients who do not respond appropriately after initiation of PAP
**Polysomnography should be considered for**
Suspicion of sleep apnea in patients with: Coronary artery disease Previous stroke or TIA Tachyarrhythmias or bradyarrhythmias Evaluation of unusual or atypical parasomnias or those with specific motor patterns To evaluate if SRBD improved after final fitting of oral appliance
**Polysomnography is an option for**
Evaluation of patients with sleep-related events, suggestive of parasomnias or nocturnal epilepsy, that are potentially injurious, have forensic implications, or insufficient response to conventional therapy Patients with intractable insomnia that have failed both cognitive behavioral therapy and pharmacological therapy

## Data Availability

No new data were created for this publication.

## Abbreviations

The following abbreviations are used in this manuscript:

DNA	Deoxyribonucleic acid
CBT-I	Cognitive behavioral therapy for insomnia
CPAP	Continuous positive airway pressure
FDA	United States Food and Drug Administration
NREM	Non-rapid eye movement sleep
OSA	Obstructive sleep apnea
PROMIS	Patient-Reported Outcomes Measurement Information System
REM	Rapid eye movement sleep
RLS	Restless legs syndrome
RNA	Ribonucleic acid
STOPBANG	Snore, tired, observed apnea, pressure (hypertension), body mass index, age, neck, gender
